# Laparoscopic Management of an Early Postoperative Pelvic Abscess Caused by Prevotella bivia Following a Deep Infiltrating Endometriosis Surgery

**DOI:** 10.7759/cureus.66315

**Published:** 2024-08-06

**Authors:** Baris Kaya, Alperen Ince, Merve Sam Ozdemir, Sercan Yuksel

**Affiliations:** 1 Obstetrics and Gynecology, Basaksehir Cam ve Sakura City Hospital, Istanbul, TUR; 2 Radiology, Basaksehir Cam ve Sakura City Hospital, Istanbul, TUR; 3 General Surgery, Basaksehir Cam ve Sakura City Hospital, Istanbul, TUR

**Keywords:** diagnostic laparoscopy in acute abdomen, prevotella bivia, deep pelvic abscess, vaginal cuff abscess, deep infiltrating endometriosis (die)

## Abstract

Surgery for deep-infiltrating endometriosis (DIE) carries a high risk of complications, including pelvic abscesses. We would like to present the laparoscopic management of a pelvic abscess caused by *Prevotella bivia* following a radical hysterectomy in a DIE laparoscopic surgery. A 43-year-old G2P2 lady underwent a laparoscopic hysterectomy, bilateral ureterolysis, bilateral parametrial nodule extirpation, and rectal shaving following complaints of severe dysmenorrhea, dyspareunia, and chronic pelvic pain due to deep-infiltrating endometriosis (ENZIAN score: P2; 02/3; T2/2; A3; B3/2; C2; FA) (American Association of Gynecologic Laparoscopists (AAGL) score: 72, Stage 4). She received intravenous antibiotic treatment at the hospital with a diagnosis of pelvic inflammatory disease one month before the endometriosis surgery. After the extensive laparoscopic surgery, the early postoperative period was uneventful; however, starting on the fourth postoperative day, she was complaining of abdominal pain. On the seventh postoperative day, severe left-sided abdominal pain, fever, nausea, vomiting, rising levels of C-reactive protein (CRP > 200 mg/dL), and signs of septicemia were observed. The vaginal examination revealed a purulent discharge. Bacterial cultures were obtained from the vaginal cuff and peripheral vein. On the computerized tomography scan, neither a bowel nor ureter injury was found, but a pelvic abscess above the vaginal cuff and left ureteral compression below the pelvic brim were observed. Due to the clinical deterioration of the patient despite receiving piperacillin/tazobactam antibiotic therapy, the decision was made to perform a repeat laparoscopy to prevent septic shock and ureteral stent application for urinary tract obstruction. During the laparoscopy, purulent fluid was discovered around the pelvic peritoneum, and it was noted that the rectosigmoid colon was edematous and tightly adherent to the pelvic sidewalls. The rectosigmoid colon was carefully detached from the pelvic sidewalls; the left ureter was released, and the purulent abscess material from the vaginal cuff was aspirated. Every effort was made to remove as many yellowish plaques covering the pelvic peritoneum and rectum serosa as possible. Recovery following surgery was rapid. *P. bivia *was detected in the blood culture, and the patient was treated with piperacillin/tazobactam for an additional seven days, resulting in a complete resolution of the illness. Pelvic abscess is a rare but serious complication that can occur following laparoscopic deep-infiltrating endometriosis surgery. To prevent ending up with septicemia and septic shock, further laparoscopic surgery may be necessary.

## Introduction

Surgery for deep-infiltrating endometriosis (DIE) carries a high risk of complications [1,2]; however, a pelvic abscess can be seen as a very rare complication. Pelvic abscess following laparoscopic hysterectomy for deeply infiltrating endometriosis was reported between 1.3% and 3% [1,3]. While most can be treated with parenteral antibiotics, with/without drainage, some need further major surgery [1,3]. Here, we would like to present a laparoscopic management of a pelvic abscess caused by *Prevotella bivia* following a radical hysterectomy of a DIE laparoscopic surgery.

## Case presentation

A 43-year-old G2P2 (VD) lady underwent a laparoscopic hysterectomy, bilateral ureterolysis, bilateral parametrial nodule extirpation, and rectal shaving with the complaining of severe dysmenorrhea (VAS score: 10/10), dyspareunia (VAS score: 6/10), and chronic pelvic pain (VAS score: 5/10) due to deeply infiltrating endometriosis (ENZIAN score: P2; 02/3; T2/2; A3; B3/2; C2; FA) (AAGL score 72, Stage 4). In her history, she was hospitalized with a diagnosis of pelvic inflammatory disease one month before the endometriosis surgery.

**Figure 1:**
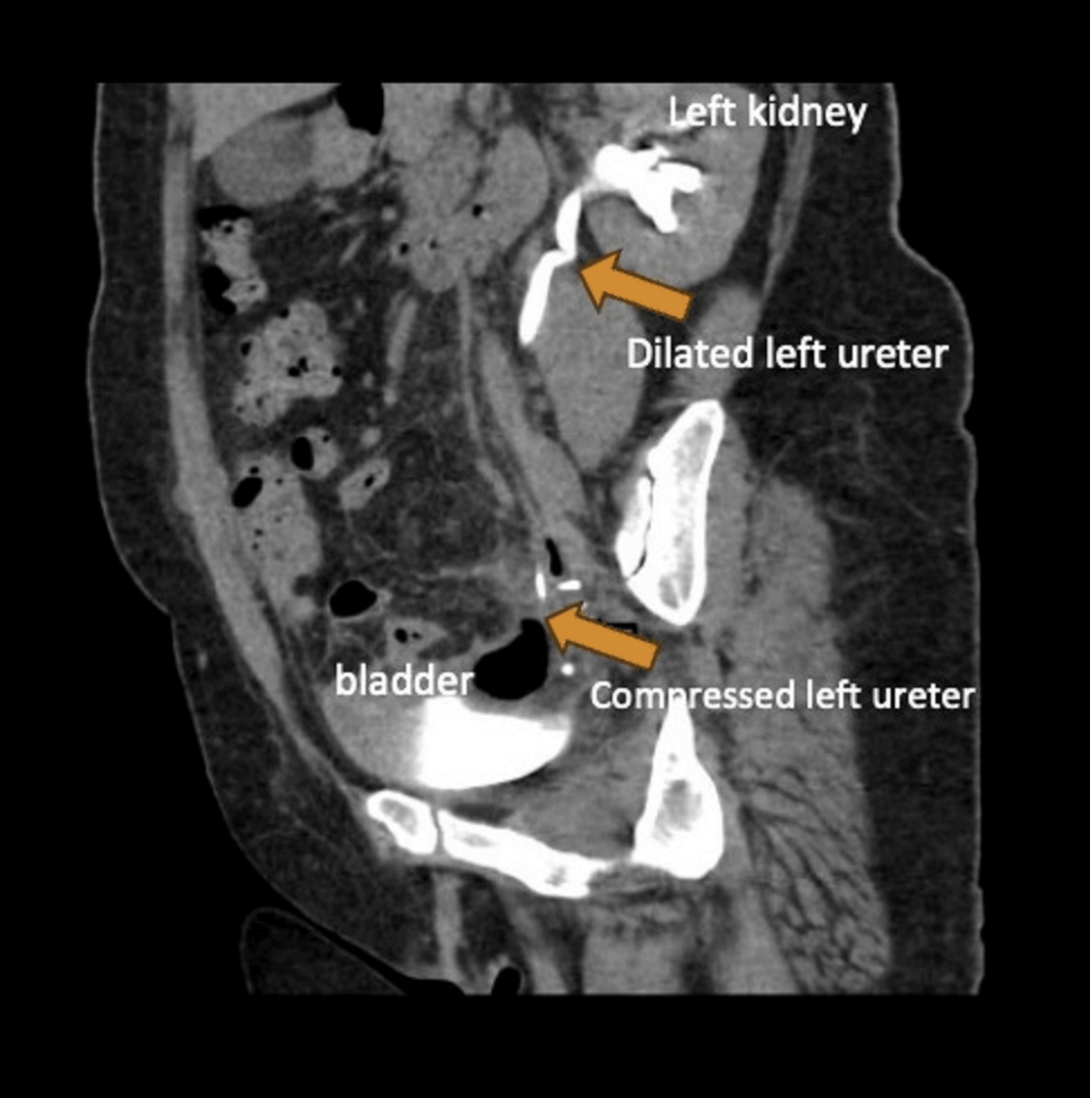
Sagittal view of left ureter compression and renal hydronephrosis in a contrast-enhanced computerized tomography scan

The early postoperative period was uneventful; the Jackson-Pratt drain was removed on the third day, and the gas-gaita discharge was positive. Discharge from the hospital was delayed due to abdominal pain, starting on the fourth postoperative day. Her complaints peaked at the top on the seventh postoperative day with severe abdominal pain, especially on the left side, fever, nausea, vomiting, and rising levels of C-reactive protein (CRP > 200 mg/dL), signs of septicemia. The vaginal examination revealed a purulent discharge. Bacterial cultures were obtained from the vaginal cuff and blood. The ultrasound revealed the presence of free fluid, particularly above the vaginal cuff. On the computerized tomography (CT) scan, neither bowel nor ureter injury was found, but a pelvic abscess above the vaginal cuff and left ureteral compression below the pelvic brim were observed (Figure 1). Due to the clinical deterioration of the patient despite receiving piperacillin/tazobactam antibiotic therapy, the decision was made to perform a repeat laparoscopy to prevent septic shock and ureteral stent application to relieve ureter compression.

During laparoscopy, purulent, free fluid was observed around the pelvic peritoneum, and the rectosigmoid colon was densely attached to the pelvic sidewalls (Video 1). First, the rectosigmoid was released from the right side of the pelvic wall, and the vaginal cuff was revealed with the purulent abscess material. The vaginal cuff, pelvic peritoneum, and rectum serosa (especially the shaved area) were covered with sticky yellowish plaques. Next, the right ureter, densely attached to the rectosigmoid colon, was released using the atraumatic forceps and aspirator with the traction-counteraction method. Continuous irrigation with sterile saline was performed to avoid organ injuries that were already infected and fragile. The rectosigmoid attached to the left pelvic sidewall was released on the left side. The rectum, which was causing compression on the left ureter, was detached. Purulent abscess material was aspirated. The infective fibrotic yellowish plaques were removed from the vaginal cuff, pelvic peritoneum, and rectum serosa as much as possible. The pelvis was washed with sterile saline, and a Jackson-Pratt drain was placed.

**Video 1:** Laparoscopic management of postoperative pelvic abscess following a deep infiltrating endometriosis surgery

Recovery was rapid following surgery. In the blood culture, *P. bivia* was detected, and the patient was treated with piperacillin/tazobactam for seven days more with complete resolution of the illness.

## Discussion

Pelvic abscess following laparoscopic hysterectomy requiring surgery is a very rare circumstance. In this case, the significant risk factor for vaginal cuff abscess was the history of hospitalization for pelvic inflammatory disease one month before the surgery. Hysterectomy for deep infiltrating endometriosis is accepted as a radical hysterectomy as the surgery extends to the parametrium, vagina, and rectum, and usually bilateral ureterolysis is performed [4]. In our case, the pelvic abscess compressed the left ureter, which was already skeletonized. In addition to the patient’s clinical deterioration, left ureter compression necessitated the surgical intervention. Another risk factor for the severity of the case was the *P. bivia* obtained in the blood culture. *P. bivia* is a Gram-negative anaerobic bacteria found in the vaginal microbiota and may be linked to bacterial vaginosis, endometritis, and pelvic inflammatory disease [5,6]. Moreover, it has been identified as the cause of severe cases of peritonitis [7] and pelvic abscess following hysterectomy [8]. Although reported cases were treated with adequate antibiotic therapy, repeat laparoscopy was mandatory due to the rapid clinical deterioration of our patient, who showed signs of severe sepsis in the early postoperative period. Koskov et al. [7] reported *P. bivia* generalized peritonitis unresponsive to broad-spectrum antibiotic therapy (gentamicin, cefazolin, metronidazole). In our case, the patient's deterioration led to the decision to perform a laparoscopy after 48 hours. The surgical procedure revealed the presence of 300 mL of purulent fluid and fibrinous adhesions in the peritoneal cavity. Additionally, slight bowel dilation, an edematous left fallopian tube, and fibrinopurulent exudate on the serosa of the appendix were identified during the surgery. The abscess material was removed from the pelvis and left salpingectomy, and an appendectomy was performed; however, contrary to our case, the surgery turned into open surgery. The patient showed complete recovery after the surgery and was treated with oral metronidazole one week after hospital discharge [7]. Sang-Min Shim and Yun-Sook Kim [8] reported a pelvic abscess following a laparoscopic supracervical hysterectomy caused by Prevotella. In their case, interestingly, the pelvic abscess occurred two months after the laparoscopic supracervical hysterectomy, which was longer than ours. The pelvic infection and cuff abscess were revealed immediately after the surgery in our case. Their case was managed medically with 14-day cefoxitin, metronidazole, and doxycycline.

Contrary to Sang-Min Shim and Yun-Sook Kim [8], our case was handled with laparoscopy due to clinical deterioration despite receiving piperacillin/tazobactam. In some cases, minimally invasive management of pelvic abscesses is necessary [9]. As reported by Koskov et al. [7], pelvic infection with *P. bivia* may be resistant to some antibiotics, and removing the abscess material from the pelvis by surgical intervention may help recovery.

The history of being hospitalized due to pelvic inflammatory disease one month before the surgery for deep infiltrating endometriosis was a significant risk factor for pelvic abscess. The patient received clindamycin and gentamycin as prophylaxis, and there was no evidence of infection during laparoscopy. It is reported that *P. bivia* can be resistant to clindamycin [10], as we have seen in our case. In this case, using metronidazole instead of clindamycin as a prophylaxis could prevent pelvic abscesses caused by *P. bivia*. Before the repeat laparoscopy, the infectious disease department changed the antibiotic therapy due to being unresponsive to clindamycin plus gentamicin. The clinical response remained unobserved, prompting the need for surgical intervention. The results of the bacterial culture were obtained after the surgery. She was treated with piperacillin/tazobactam one week after the operation for complete recovery.

Pelvic abscess following endometriosis surgery might be severe compared with benign gynecologic hysterectomy due to extensive surgical removal of the nodules from the parametrium and other pelvic organs, such as the rectum. Further, early surgical management with minimally invasive gynecology has advantages, such as high success and low readmission rates [11], as proven in our case.

## Conclusions

Pelvic abscess is a rare but serious complication that can occur following laparoscopic deep infiltrating surgery and may require further laparoscopic surgery. In our case, laparoscopic management of the pelvic abscess was successful and prevented septic shock. Historically, surgical management of abscesses has repeatedly proven its effectiveness.
